# Supplementary material for: Garden centre customer attitudes to pollinators and pollinator-friendly planting
Source: PeerJ. 2019 Jun 7;7:e7088. doi: 10.7717/peerj.7088 (PMC6557251; doi:10.7717/peerj.7088)
Supplement: Supplemental Information 1 — (A) Survey results, (B) customer questionnaire, (C) customer information sheet and (D) semi-structured interview format. [file peerj-07-7088-s001.docx]

Appendix A: Survey Results

| Question | Answer | % overall (n=150 unless otherwise stated) | % Male (n=32) | % Female (n=118) |
| --- | --- | --- | --- | --- |
| 1. What is your age? | 16-25 | 2.0 | 0.7 | 1.3 |
|  | 26-35 | 2.7 | 1.3 | 1.3 |
|  | 36-45 | 2.7 | 0 | 2.7 |
|  | 46-55 | 14.7 | 1.3 | 13.3 |
|  | 56-65 | 26.7 | 4.0 | 22.7 |
|  | 66+ | 51.3 | 14.0 | 37.4 |
| 2. What is your gender? | Male | 21.3 | - | - |
|  | Female | 78.7 | - | - |
|  | Prefer not to say | 0 | - | - |
| 3. Do you have an outside space in which to grow plants? *Please tick* ***all that apply****.* | Yes, a garden | 94.7 | 20.0 | 74.7 |
|  | Yes, an outside space *(eg. communal space, allotment, window boxes, pots)* | 8.0 | 2.0 | 6.0 |
|  | No | 0.7 | 0 | 0.7 |
| 4. What is the purpose of your visit today? | By plants or seeds/bulbs | 56.7 | 11.3 | 45.3 |
|  | Buy other gardening items *(eg. tools, fertiliser, garden furniture)* | 22.0 | 4.7 | 17.3 |
|  | Buy other items not connected to gardening/gardens *(eg. a gift, food items)* | 16.0 | 3.3 | 12.7 |
|  | Leisure *(eg. having a browse, meeting at the café)* | 52.0 | 9.3 | 42.7 |
| 5. When you are buying plants do you most often look for: *Tick* ***one box*** (n=138) | Ornamental plants with flowers *(e.g. bedding plants/perennials)* | 68.1 (n=138) |  |  |
|  | Vegetable/fruit plants | 7.2 |  |  |
|  | Trees or shrubs | 19.6 |  |  |
|  | Indoor plants | 5.1 |  |  |
| 6. When you are buying flowering plants, what do you generally consider the **three** most important features **apart from price**? *Tick* ***three boxes*** (n=145) | Length of flowering | 55.1 (n=145) |  |  |
|  | Hardiness/low level of maintenance | 55.9 |  |  |
|  | Attractiveness to humans | 22.8 |  |  |
|  | Bee- or Pollinator-friendly | 53.1 |  |  |
|  | Specific colour | 26.9 |  |  |
|  | Size | 21.4 |  |  |
|  | Time of year when it blooms | 43.4 |  |  |
| 7. Does the decline of wildlife in Britain concern you? | Yes | 97.3 |  |  |
|  | No | 1.3 |  |  |
|  | I didn’t know that our wildlife was in decline | 1.3 |  |  |
| 8. Do you do anything in your garden or outside space to help wildlife? | Yes | 96.7 |  |  |
|  | No | 3.3 |  |  |
| 9. Thinking about bees and other pollinators, do you think they are beneficial to your garden or outside area? | Yes | 97.3 |  |  |
|  | No | 1.3 |  |  |
|  | I don’t know | 1.3 |  |  |
| 10. How do you help bees and other pollinators in your outdoor area? *Tick* ***all that apply to you*** | Put up ‘bee hotels’ | 19.3 |  |  |
|  | Use limited or no pesticides | 64.0 |  |  |
|  | Grow bee-friendly and pollinator-friendly plants | 77.3 |  |  |
|  | Provide flowers throughout the year | 57.3 |  |  |
|  | Leave some areas unmown/unmanaged | 37.3 |  |  |
|  | I don’t currently help bees/pollinators | 2.7 |  |  |
| 11. Do you know that some flowers are much more attractive to bees than others? | Yes | 98.7 |  |  |
|  | No | 1.3 |  |  |
| 12. Are you familiar with ‘pollinator-friendly’ labels, such as the RHS “Perfect for Pollinators”? | Yes | 59.3 |  |  |
|  | No | 40.7 |  |  |
| 12(i). Are you familiar with ‘pollinator-friendly’ labels, such as the RHS “Perfect for Pollinators”? (n=53, *subset of 53 people who thought garden centres offer enough information about which plants are good for pollinators [Q16: Yes]*) | Yes | 71.7 (n=53) |  |  |
|  | No | 28.3 |  |  |
| 13. Do you think these labels are reliable sources of information | Yes | 70.7 |  |  |
|  | No | 2.7 |  |  |
|  | I don’t know | 26.7 |  |  |
| 13(i). Do you think these labels are reliable sources of information (n=89*; subset of 89 people who were familiar with pollinator-friendly labels [Q12: Yes]*) | Yes | 87.6 (n=89) |  |  |
|  | No | 1.1 |  |  |
|  | I don’t know | 11.2 |  |  |
| 14. If a plant has a ‘pollinator-friendly’ label would you be more inclined to buy that plant? | Yes | 64.0 |  |  |
|  | No | 4.0 |  |  |
|  | Maybe | 32.0 |  |  |
| 15. Do you think the labels are visible enough? | Yes | 51.3 |  |  |
|  | No | 48.7 |  |  |
| 15(i). Do you think the labels are visible enough? (n=89; *subset of 89 people who were familiar with pollinator-friendly labels [Q12: Yes]*) | Yes | 74.2 (n=89) |  |  |
|  | No | 25.8 |  |  |
| 16. Do you think garden centres offer enough information about which plants are good for bees and other pollinators? | Yes | 35.3 | 8.7 | 26.7 |
|  | No, and it would be useful to have more information | 50.0 | 10.0 | 40.0 |
|  | No, but I don’t mind | 2.0 | - | 2.0 |
|  | I don’t know | 12.7 | 2.7 | 10.0 |
| 17. Do you ever ask the staff for advice about suitable plants for bees and other pollinators? | Yes | 23.3 | 4.7 | 18.7 |
|  | No | 76.7 | 16.7 | 60.0 |
| 18. Any further comments about pollinator-friendly plants | ………. | … | … | … |

Appendix B: Customer Questionnaire

Appendix C: Customer Information Sheet

Appendix D: Semi-Structure Interview Format

**Semi-structured interview – Garden Centre customers**

*Researcher:* Before starting this interview, I’d like to make it clear that I will be recording our conversation. Is that ok?

--------------------------------------------------------------------------------------------------------------------------

**Section 1:** *Preamble and survey*

[A]

1. Roughly how often do you visit garden centres?
2. Would you say this is the centre you normally visit?

[B] *Customer Survey*

*Researcher:* Please answer the Customer Survey with no input from me/the researcher. This should take roughly 3-5 minutes.

--------------------------------------------------------------------------------------------------------------------------

**Section 2:** *Based on questions from customer survey*

*Researcher:* Looking at your responses to the Customer Survey, there are a few questions I’d like to explore in more detail.

6) When you are buying flowering plants, what do you generally consider the **three** most important features **apart from price**?

*Researcher:* Could you tell me a little about why you selected these options?

8) Do you do anything in your garden or outside space to help wildlife?

*Researcher:* Could you tell me in brief how you help wildlife in your outside area? Is there any specific wildlife you particularly like to encourage - why?

9) Thinking about bees and other pollinators, do you think they are beneficial to your garden or other outdoor area?

*Researcher:* [If yes] Why/how? [If no] Why not?

11) Do you know that some flowers are much more attractive to bees than others?

*Researcher:* [If yes] Do you know why this is?

12) Are you familiar with ‘pollinator-friendly’ labels, such as the RHS “Perfect for Pollinators”?

*Researcher:* I’ve got a sheet here with some labels on [see supplementary materials]. Could you tick the boxes next to all the logos you recognise or are familiar with?

13) Do you think these labels are reliable sources of information?

*Researcher:* Any further comments about your answer to this question?

14) If a plant has a ‘pollinator-friendly’ label would you be more inclined to buy that plant?

*Researcher:* Why have you answered yes/no/maybe?

15) Do you think the labels are visible enough?

*Researcher:* Any further comments about your answer to this question?

16) Do you think garden centres offer enough information about which plants are good for bees and other pollinators?

*Researcher:* Regarding your answer to this question, why have you responded with XXX?

--------------------------------------------------------------------------------------------------------------------------

**Section 3:** *Exploring further topics*

*Researcher:* Following on from the Customer Survey, there are a couple of related questions I’d like to ask.

1. Has your awareness of/interest in bees and other pollinators/pollinator-friendly plants changed over time? If so, could you tell me a little more about this?

2. Where do you think you receive most information about pollinators?
